# Patient and public involvement in patient safety research: a workshop to review patient information, minimise psychological risk and inform research

**DOI:** 10.1186/s40900-016-0035-x

**Published:** 2016-05-17

**Authors:** Dominic Furniss, Ioanna Iacovides, Imogen Lyons, Ann Blandford, Bryony Dean Franklin

**Affiliations:** 1 UCL Interaction Centre, University College London, Gower Street, London, UK; 2 Centre for Medication Safety and Service Quality, Imperial College Healthcare NHS Trust, London, UK; 3 Research Department of Practice and Policy, UCL School of Pharmacy, Mezzanine Floor, BMA House, Tavistock Square, London, UK

**Keywords:** Patient and public involvement, Patient safety, Medication error, Intravenous medication, Health services research

## Abstract

**Plain English summary:**

Patient safety is a growing research area. However, although patients and the public are increasingly involved in clinical research, there is little guidance on how best to involve patients in patient safety research. Here we focus on how patients can contribute to the design of patient safety research.

We conducted a workshop with patients as part of a project exploring errors and safety in the delivery of intravenous medication (medication given via a vein). The workshop was designed to explore how best to engage with hospital inpatients about these issues, to generate research topics, and to inform researchers about patients’ experiences. Nine patients participated, each of whom had previously received intravenous medication. Participants advised against using terms such as ‘error’; they also advocated caution when using terms such as ‘safety’ when describing the study to patients as this may worry some who had not thought about these issues before. We received thorough and useful feedback on our patient information sheets to ensure they were clear and understandable to patients. Patients also shared rich experiences with us about their treatment, which emphasised the need to extend our research focus to include a wider range of factors affecting quality and safety.

**Abstract:**

**Background**

Patient safety has attracted increasing attention in recent years. This paper explores patients’ contributions to informing patient safety research at an early stage, within a project on intravenous infusion errors. Currently, there is little or no guidance on how best to involve patients and the wider public in shaping patient safety research, and indeed, whether such efforts are worthwhile.

**Method**

We ran a 3-hour workshop involving nine patients with experience of intravenous therapy in the hospital setting. The first part explored patients’ experiences of intravenous therapy. We derived research questions from the resulting discussion through qualitative analysis. In the second part, patients were asked for feedback on patient information sheets considering both content and clarity, and on two potential approaches to framing our patient information: one that focused on research on safety and error, the other on quality improvement.

**Results**

The workshop led to a thorough review of how we should engage with patients. Importantly, there was a clear steer away from terms such as ‘error’ and ‘safety’ that could worry patients. The experiences that patients revealed were also richer than we had anticipated, revealing different conceptions of how patients related to their treatment and care, their role in safety and use of medical devices, the different levels of information they preferred, and broader factors impacting perceptions of their care.

**Conclusion**

Involving patients at an early stage in patient safety research can be of great value. Our workshop highlighted sensitivities around potentially worrying patients about risks that they might not have considered previously, and how to address these. Patient representatives also emphasised a need to expand the focus of patient safety research beyond clinicians and error, to include factors affecting perceptions of quality and safety for patients more broadly.

## Background

### Patient safety and the role of patients

Patient safety is a global health issue [27]. The Institute of Medicine’s report *To Err is Human* estimated that between 44,000 and 98,000 Americans die each year due to medical error [13]. Even assuming the lower estimate, this suggests it is the eighth leading cause of death in America, with more people dying from medical error than motor vehicle accidents every year. Similarly grim statistics are contained within the NHS report *An Organisation with a Memory,* which estimates that around 1 in ten patients admitted to NHS hospitals will experience an adverse event [5]. Among these statistics are real stories of human tragedy. Increased awareness of unacceptable levels of preventable patient harm has led to global efforts to improve patient safety and increased research in this field [27, 28].

Despite patients being the main stakeholder in their care, their potential contribution to patient safety was largely ignored until the turn of the millennium [26]. Such contributions could help with reaching an accurate diagnosis, choosing appropriate treatment plans, monitoring treatment and conditions, recognising and reporting adverse events, improving how incident reporting is handled, and putting pressure on policy makers to improve standards [15, 26]. The patient’s potential contribution is particularly important because they and their families are the only people present along the entire continuum of care in which they engage with multiple healthcare providers from different disciplines, and sometimes from different facilities [27].

Patient participation and involvement in patient safety is sometimes seen as a single broad area [14]. However, it is important to distinguish between patients and the public participating in safety practices and involving patients in research on patient safety. For example, the former would include a patient confirming which limb a surgeon will operate on, whereas the latter would include patients advising researchers on the design of research studies. Previous research has investigated the role of patients in their own care and the co-production of patient safety in practice (e.g. [6, 10, 26]), but there is little published work on the patient’s role in shaping patient safety research.

### The patient’s role in shaping patient safety research

With respect to the patient’s role in research activities, INVOLVE [11] outline the different types of role that patients and the public can have:

- Participation - the traditional role of patients within research where they are the subject of research and provide data to be analysed.
- Involvement – lay people actively working with researchers who seek help and advice on the design, management and/or conduct of research, which can also include their active involvement in data gathering and/or data analysis.
- Engagement - dissemination of research findings and their implications to patients and the public.

In contrast to patient participation in their own safety, patient involvement in research focuses on helping to shape the research, e.g. prioritising research topics, informing how studies should be conducted, actually conducting data gathering and analysis, and giving advice on how to conduct engagement.

Current work on patient and public involvement (PPI) in research suggests there are few practical examples of how it can be conducted and its potential benefits [12]. Some positive PPI examples have been documented in response to threats of tokenism [24], which also include details of the ethical issues and practicalities of using community researchers and patients to gather data in hospital [9, 21]. These studies demonstrate the value of PPI in their qualitative descriptions of context, experiential knowledge and contributions to the project [23].

Recognising the special sensitivities in patient safety research, the World Health Organization published advice on *Ethical Issues in Patient Safety Research* [28]. The absence of any reference to patient and public involvement (PPI) activities in this is notable. For example, they advise that a safety committee of expert clinicians could be set-up to advise on how to handle sensitive issues that arise during the conduct of the study, but they do not mention how PPI could also advise on ethical issues and how they can be addressed before the study begins. We explore the role PPI has to play in the early stages of patient safety research by 1) reviewing patient safety information and psychological risk; and 2) informing research topics and sensitizing researchers to patients’ experiences. These aims were shaped by early PPI feedback on our proposal for a project to explore errors and safety in the delivery of intravenous medication.

### Reviewing psychological risk and patient safety information

When initially developing our research proposal, we sought informal feedback on our project plans from two patient representatives. One was a patient representative from an earlier related project and the other an acquaintance who had recently been an inpatient, both of whom had received intravenous therapy.

The feedback received at this stage raised potential issues about the conduct of the study. For example one of the patient representative giving feedback on our proposal said “*…for patients and relatives [the proposed project] contains too much information regarding hazards. The patients and relatives need to have complete confidence in the staff and equipment, almost blind faith in many instances*”. This caution against exposing patients to details about potential errors was in stark contrast to our desire to inform potential participants about the study in an open manner. We therefore wanted to explore how to approach patients about patient safety issues while minimising psychological risk. Psychological risk refers to the potential to have detrimental effects on the psychological state of the participant, e.g. raising concerns, anxieties and levels of stress.

The WHO [28] guidance notes: “*Psychological risks include the possibility that research participants will become emotionally distressed, fearful or anxious as a result of their participation. For example, studies that interview patients or families about harmful incidents that occurred previously or about their perceptions of the quality of care may cause them to question the quality of their medical providers and to become anxious.*” WHO ([28] p.15). To support this, Rhodes et al. [20] found that, during their research, many patients had not thought about any safety issues in depth until they were asked. One area where PPI could make a positive contribution to patient safety research is to review potential psychological risks and mitigating strategies from a patient’s perspective before the study begins. This has not been reported on before.

Typical PPI activities include reviewing patient information so it is written in plain English and more easily understood from a patient’s perspective. However, there are not many published accounts of this in practice, and no accounts of the special sensitivities associated with patient safety terminology. In their work with primary medical care patients Rhodes et al. [20] deliberately avoided framing their research questions in terms of ‘error’ and ‘harm’ and let patients introduce topics they considered relevant. However, published accounts of PPI advice in this area is lacking.

### Informing research topics and sensitizing researchers to patients’ experiences

The initial informal patient feedback received on our project plans also encouraged us to investigate intravenous infusion practices from the patient’s perspective. For example, one of the patients providing feedback informed us that, “*patients have no idea of what is going on. […] I was told next to nothing about what was going on.*” We proposed to involve patients as participants in our research to investigate experiences of their intravenous infusion practices, both positive and negative. However, before doing that we wanted to use PPI to help focus our research questions and sensitize ourselves, as researchers, to patients’ experiences in this area.

Sensitizing researchers to patients’ experiences works at two levels: 1) by informing the researcher’s knowledge of an area so that they are more aware of potential issues, and 2) by informing the researcher’s emotional awareness and the experiential aspects associated with speaking to patients around the topic of research.

As part of helping to focus our research questions we were interested to hear from our PPI participants about their general experiences of receiving intravenous medication including, but not limited to, the extent to which patients were involved in intravenous infusion administration. For instance, in previous studies we had observed an indirect role for patients as a member of staff realised a mistake as they explained the intravenous medication details to a patient [7], and a more direct role as patients silenced alarms of their infusion pumps [8]. Although the role of patients in patient safety has been investigated generally (e.g. [3, 4, 6, 10, 14, 17–19, 22, 26]) we know of no published accounts in the specific area of intravenous medication infusion.

In this paper we report on a detailed case study of a PPI workshop that has informed the design of our patient safety research. This is the first detailed published account of patient involvement in shaping patient safety research. We provide a description of our PPI activity, report experiential knowledge gained through the process, and document the PPI contribution to our project. Specifically we believe that a PPI workshop can help to review patient information and psychological risk, inform research topics and sensitize researchers to patients’ experiences.

## Method

### Study setting

Our PPI workshop took place in the context of a larger project called ECLIPSE (Exploring the Current Landscape of Intravenous Infusion Practices and Error) [1]. ECLIPSE seeks to investigate different intravenous infusion practices in English hospitals and how these relate to the prevalence of error, in order to make recommendations for safer infusions.

ECLIPSE has an eight-member steering committee, one of whom is a patient representative, as well as a wider advisory group with twelve members, two of whom are patient representatives. The three phases of research in ECLIPSE, and their associated PPI activities, are shown in Table 1. The workshop described in this paper is part of the first phase, with the workshop being viewed as a preliminary activity to inform the planned research.

**Table 1:** Planned activities related to the three phases in ECLIPSE

Phase	Research methods	Patient involvement, participation & engagement
1) Point-prevalence study	Quantitative observational point-prevalence study of intravenous infusions, at 16 hospital sites In-depth discussions with key staff at participating sites to understand practices	First patient and public involvement workshop to review patient information sheets for Phases 1 and 2; and explore potential topics for investigation in Phase 2
2) In-depth observational study	Follow up ethnographic study of a subsample of participating sites to explore practices in more detail	Interview patient participants on wards about their intravenous infusion experiences at different sites
3) Recommendations and summary reporting	Dialogue with participating hospitals to jointly identify and communicate recommendations for best practice	Second patient and public involvement workshop to reflect on the results and discuss dissemination

### Objectives of the workshop

A PPI workshop was designed to address two main objectives:

1. Establish how to appropriately inform and engage potential patient participants in our research.
2. Sensitise researchers to patients’ experiences, and shape research topics and questions related to intravenous infusions practices and safety for Phase 2 of the study.

### Recruitment and sampling

Information about the focus of the workshop, date, time, location, compensation, requirements for attendance, and links to the wider project were posted on the project’s website (http://www.eclipse.ac.uk) and People in Research (http://www.peopleinresearch.org), a website that advertises PPI opportunities. We also circulated the relevant information to existing contacts. Experiencing harm was not a pre-requisite to participate; indeed we wanted ‘normal’ experiences to be the focus. Six patient representatives were recruited via these different channels. The three patient representatives on our steering committee and advisory group also participated. These patient representatives had seen our project plan and so were familiar with the high-level objectives of the research, but they had no involvement in organising the workshop and were largely unfamiliar with the researchers when it was held.

All nine attendees (2 male, 7 female; approximate age range 35–70) had first-hand experience of receiving intravenous medication in hospital, and some had additional experiences of friends and family receiving intravenous medication. One attendee was also a qualified nurse. Some patients were familiar with patient and public involvement activities, e.g. the steering committee member had experience of reviewing patient information material and someone entirely external to the project had experience of sitting on Research Ethics Committees.

### Procedure

The first three authors ran the workshop on university premises. Their research interests include medication safety, the psychology of human error and human factors (i.e. evaluating and designing products and systems while taking proper account of the people who use them). The 3-hour workshop was organised into five parts:

1. Introduction to the workshop;
2. Patients’ experiences with intravenous infusions (in two sub-groups)
  [lunch break];
3. Reporting back from the two sub-groups;
4. Review of ECLIPSE patient information sheets and discussion about how best to engage with patient participants;
5. Wrap-up session.

During the introduction we outlined the scope and plan for our project, and the role of the PPI workshop within it. As researchers, we recognised the benefits of a bottom-up approach to exploring patients’ experiences in an open way - generating research topics and themes without restriction. However, we were aware that this had to be balanced with trying to remain within the remit of the project. For example, we were conscious that it could be considered unethical to create expectations about researching areas that were outside the project’s remit. Participants were therefore informed about this challenge and the project’s constraints so they could help us manage this issue.

We intentionally started by listening to patients’ experiences of receiving drugs and fluids intravenously as this would set the context for the rest of the workshop. Given the size of the workshop, i.e. nine patients and three researchers, we split the participants into two parallel groups to allow for in-depth discussion. There were four patients and one researcher in one group, and five patients and two researchers in the other. The division was made arbitrarily. Each group had a researcher with experience of facilitating focus group discussions. The lunch break was also strategically placed so that conversations could continue over lunch before reporting back to the whole group on the main topics of discussion.

In the second part of the workshop, patients were asked to provide feedback on the patient information flyer for Phase 1 of ECLIPSE and what the Phase 1 observers should say to patients (i.e. we had written a small introductory paragraph for Phase 1 observers to use about the project). We were not involving patients as participants in Phase 1 and so only needed a flyer to inform them about the study taking place on the ward. The Research Ethics Committee had already approved these. The attendees were then given two alternative patient information sheets for Phase 2, which would be used to seek informed consent for patient participation. One informed the patient about ECLIPSE and the study’s aims of reducing error, and the other was framed around quality improvement. Patients were asked about their preference and to suggest improvements. Documentation for Phase 2 had not been submitted to the Research Ethics Committee at the time of the workshop.

### Ethical considerations

As this was a PPI activity to shape research, rather than an activity involving patient participation in research, NHS research ethics approval was not required. However, the UCL Research Ethics Committee granted ethical approval (UCLIC/1213/023/Staff) for audio recording of the discussions and use of this in subsequent analysis. Patient representatives were given information sheets to describe what was involved in participating in the workshop, its objectives, data protection, compensation and reimbursement. Each signed to confirm their consent. We sought ethical approval for this involvement activity for two reasons: 1) this provided confirmation that we were in line with good research practices such as fully informing the workshop participants as to what was involved; and 2) to obtain consent for audio-recording the workshop discussion and using the data in publications. A similar rationale for seeking ethical approval for a PPI activity has been reported elsewhere [16].

### Data collection

All workshop discussions were audio recorded and subsequently professionally transcribed. The researchers also took notes and annotated patient information sheets to capture patients’ feedback.

### Data analysis

The transcripts and notes were reviewed. Codes and themes were identified inductively using principles of thematic analysis [2]. Through carrying out the analysis in this way we were able to approach the data collected in a systematic manner, thus increasing the integrity of our analysis, which would inform our research. The resulting data set and findings could also be shared more easily across the research project team. The first author performed the main analysis. The second and third authors reviewed the transcript, codes and resulting themes to identify any themes that had been overlooked. QSR NVivo version 10 was used to facilitate this process.

## Results

The findings are presented according to our workshop objectives: 1) how to appropriately inform and engage patients in our patient safety research; and 2) sensitising researchers to patient experiences and exploring topics that will be further investigated through research in Phase 2 of the project.

### Informing and engaging patient participants

Two main issues were highlighted in terms of how to inform patients about the research. The first was simplifying the patient information provided, and the second was the potential for raising concerns among patients about errors or poor care.

### Feedback on phase 1 flyer: simplifying patient information

The information flyer for Phase 1 had been reviewed internally within the project team, and approved by an NHS Research Ethics Committee. However, participants expressed concerns about both the length of the flyer, and the language used to convey information about the study and taking part. They emphasised the need to reduce the burden on participants, recognising that they may be very unwell when receiving this information.

Patients drew our attention to particular words and phrases that could be simplified. For example, most patients agreed that the term ‘drip’ would be better than ‘intravenous medication’, and that asking patients about ‘using’ the pump would be more easily understood than ‘interacting’ with it. The information sheet also specified that patient names and hospital numbers would be ‘disposed of’ following data collection, which they thought was a poor choice of phrasing.

Patients wanted a shorter and simpler flyer; however, one patient who had Research Ethics Committee experience recognised that the longer format is what would be expected. The group also questioned the necessity of the legal note: “NHS Indemnity does not offer no-fault compensation i.e. for non-negligent harm, and NHS bodies are unable to agree in advance to pay compensation for non-negligent harm.” This was described as ‘gobbledygook’, but again the group agreed that this probably needed to be included if this was a standard phrase.

### Feedback on phase 2 information sheets: concerns about safety and compromising care

To generate discussion about how to appropriately engage with patients in Phase 2 we provided patients with two potential versions of an information sheet. Sheet A was framed around ECLIPSE’s focus on “understanding and reducing the prevalence of medication error,” and sheet B was framed around “developing strategies to improve safety.” Sheet A was quickly dismissed as unworkable as there was broad agreement that mentioning the term ‘error’ could alarm patients. Sheet B was preferred; however, some patients were also concerned about use of the term ‘safety’:

*Patient 1: “…immediately you flag up the word safety and you’ve got people worrying.”*

Further discussion suggested that safety terms could be used, but they needed to be used with care. Patient 8 said they would be comforted to know that this work was going on and that safety was being checked. However, there was some recognition that while this might suit some patients, it might not be comforting to others. In summary, workshop participants were broadly supportive of the approach used in sheet B with an emphasis on quality improvement and improving safety rather than reducing error.

In addition, participants suggested that ‘poor’ should be deleted in the phrase “action will be taken if researchers have concerns about poor practice” which was initially intended to reassure patients but they thought it could raise concerns. They also did not think the phrase ‘action will be taken’ was informative and friendly. This resonates with existing research as patient do not want to engage in activities that can be seen to be ‘checking up’ because they have relationships to manage between themselves and their healthcare providers (e.g. [10]). To reassure patients who did not want to take part, the draft information sheets stated, “This would not affect the standard of your care.” However, there was some concern about how this statement could be interpreted and participants suggested improvements:

Patient 6: *“The other thing that I would worry about, to a degree, is the sentence, this would not affect the standard of care you receive. […] I wouldn’t be sure whether that was a threat.*

Patient 1: *You have to say that, though, don’t you?*

Patient 4: *You could word it to say you’ll get the same care you always would.*

Patient 5: *Yes, it’s about reassuring people that if they drop out, they’re not – there’s not going to be any penalties for it, essentially, but it’s a horrible way of putting it. […] It’s one of those things that raises more questions than it answers.”*

Some also raised concern about observers ‘checking’ their prescription as this could imply that there may be something wrong. Further, participants were keen to convey that this was a broader study across the whole ward and the hospital so patients did not feel their particular care was being singled out for any reason.

### Informing research topics and sensitising researchers to patients’ experiences

Issues that emerged from patients sharing their experience of intravenous infusions have allowed us to draft a list of questions to consider for the patient interviews in Phase 2, which are broader in scope than our initial ideas and grounded in patient experiences:

- What diversity is there in the way infusions are administered and what factors influence the patient’s perception of the quality of their care?
- To what extent do infusion practices instil patients with confidence? Do they get the level and type of information they want? Do they understand enough about their intravenous treatment?
- What issues do patients have with their intravenous medication administration and infusion pumps?
- What does patient participation look like in the context of intravenous medication administration? What factors affect patient willingness to participate in safety behaviours related to their infusions? How interested are patients in their pumps? Do patients interact with their own pumps, and under what circumstances?
- What information about intravenous infusions do patients think would be useful to provide other patients? What would be the best way to share this information, e.g. a leaflet?
- How could intravenous infusion practice be improved from a patient’s perspective?

To demonstrate how these questions emerged from the workshop we highlight some of the topics discussed using direct quotations to preserve the patient’s voice.

Participants reported a wide diversity in the way infusions were administered and what influenced the quality of their care. For example, one patient complained about the lack of information received from some staff in the emergency department, despite asking:

Patient 7: “*I kind of found it quite impersonal, to be honest with you, the approach of the nurses. […] It wasn’t really explained. […] when I asked what was going on and why it was being flushed with water, the answer that was given was kind of grudgingly given, as if to say, well, what’s it to do with you, you know. We’re in charge here.*”

In contrast another patient felt that her care team involved her in her care, and kept her as happy and engaged as they could, which was critical to her recovery due to the extent of her illness and her extended isolation:

Patient 2: “*In fact, during my 10 months seeing only healthcare professionals was actually really interesting because we cracked a lot of jokes, [and] some people might think they were theatrical, they were kind of trivial, not important, but in fact, they’re very, very important to keep the patient in focus, especially when the patient is very ill, to keep the patient as involved as possible.*”

A different patient highlighted that the attitude of staff could reveal itself and impact their perception of care through indirect means; e.g., throwaway comments and discussions between staff, when they think patients cannot hear, can have a big impact on their confidence in their care:

Patient 6: “*There was a nurse […] post-surgery, I was in the ICU for 48 h or something and then shipped up to the ward for, I can’t remember the phrase now, but specialised nursing. And so there was two nurses there for something like 48 h constantly […]. You know, lots of drips and infusions and pain relief. And one of them, obviously, was really annoyed that, as far as she was concerned, that she had to sit in a special wing. I mean, I was fairly well conscious by this time, but still, you know, morphine going in, so you’re sort of… so a bit cloudy and a bit vague […] and you can hear all these comments. And she’s teaching another nurse and she was saying, you know, we shouldn’t be doing this. We should be somewhere else in another ward, in a main ward, not in a side room like this. This is special treatment.*”

Patients empathised with staff who often had to deal with difficult jobs in difficult circumstances, and who may be having a bad day, but they also recognised the need to be professional and thought that some staff just did not have the right attitude for the job. Patients also reported good experiences and were full of admiration for staff who contributed to their care and went out of their way to make them feel comfortable:

Patient 1: “*If you think they know what they’re doing and they really want to help you, you feel much more relaxed and much happier about them attaching things to you and pumping things into you and pumping them out. And the really good ones do explain things. [One positive experience involved a member of staff waiting with me for my chemotherapy to finish long beyond the end of his shift.] And he didn’t moan at all. We had a long chat about holidays and where he came from [and] he just made me feel that I wasn’t being a nuisance. […] And when you’re so anxious about the whole thing, it makes such a difference.*”

The provision of information can affect patient understanding. In one case a patient given a patient-controlled analgesia pump for pain relief did not know how often she could press the button and whether she could overdose. Patients also remarked on not knowing if air in the line is a problem for them to worry about.

Patient 1: “*I was going to say, you don’t know whether you should be panicking about air in the line, do you, because as a patient, people say, you can get air in and you’ll be dead, so that’s the modern view of it – that’s the modern myth. I don’t know to what extent it’s true but that’s the perception people have […]*”

Patients also did not know why pump alarms were going off.

Patient 3: “*It’s that understanding of what and why and [Patient 5] very rightly corrected me. I said pumps go off for no reason and they go off for a reason because something’s wrong. I think [there is a] difference [between] the repetitive alarms when they’ve just been silenced [and] actual error alarms. I think that’s the patient safety issue. It’s not knowing whether [staff silence the alarms because they don’t have time to attend to actual error alarms or whether the alarm is for some sort of repetitive alert.]*”

Patient 1: “if you’re a patient, you don’t speak pump beep, beep.”

Some patients would not dream of touching their pumps whereas others, particularly those in hospital for a long time, learnt how to use theirs to some degree.

Some participants expressed concern about their intravenous treatment being set up properly but felt it was hard to question processes they did not understand fully and in some cases were concerned about undermining the healthcare professional by asking questions.

Participants suggested developing a leaflet or poster or similar to improve understanding around intravenous infusions and pumps, to make people less frightened and empower patients to ask questions. Participants also raised the challenge of adapting information to the different needs of patients and their different reactions to it.

Participants were aware that the quality of staff, equipment and staffing levels would affect their care. The maintenance and availability of equipment was raised as a specific issue. Patients were aware of broken equipment being put aside, and shortages of equipment that needed to be borrowed from adjacent wards, which did not instil confidence in the equipment that they were relying on.

Patient 2 commented on how some staff treat the equipment “*what I found with the equipment is that in the case of some nurses, not all, but some nurses, they don’t care. They have no attention to detail, they don’t care about the equipment and so the equipment isn’t working very well.*”

The quality of equipment was brought up as a different issue as one participant felt that kinks in giving sets could be more prevalent or problematic with cheaper products.

## Discussion

Staley [23] argues that we need more details of PPI activity to understand ‘how it works’ and what value it adds. In the following sections we outline the main contributions of our PPI workshop in shaping the design of patient safety research.

The format of the workshop, which ran like a focus group, proved to be successful. Patients were able to compare and contrast their experiences as stories were shared and discussed in the group. This provided common themes as well as a rich source of variability. It may not be appropriate to discuss all patient safety topics in a group format, e.g. particularly sensitive topics might be more appropriate on a one-on-one basis. However, patients with shared experiences are likely to be able to better empathise with one another.

### Reviewing potential psychological risk and mitigating strategies for patient safety research through PPI

The WHO’s [28] guidance on ethical issues for patient safety research draws attention to the potential psychological risk as participating patients could be worried about possible errors. Although the WHO [28] guidance lacks any mention of PPI activities, our workshop shows that PPI can make a positive contribution to addressing this issue in patient safety research. Patient representatives can review research plans, anticipate how patients might feel, and suggest mitigating strategies.

The initial PPI feedback we received when drafting the project proposal suggested our project could increase anxiety in patients due to the issues it raises. We therefore explored this issue in the workshop. Patients gave a clear steer away from terms such as ‘error’ and even warned that terms such as ‘safety’ should be used with care. Others have adopted similar approaches, e.g. Rhodes et al. [20] avoided the use of ‘error’ and ‘harm’ in their interviews with patients. This does not mean that we should not provide information about risks, but that we need to do so in an open and honest way that manages patient anxiety. Patients also raised other areas of concern with our patient information material that we had not foreseen, which could be used in other patient safety projects, e.g. to ensure that patients did not feel that they, or their care, were being singled out for any reason.

### Simplifying patient information

Reviewing patient information material is seen as a fairly standard function of patient and public involvement; however, we have not seen many publications on this – perhaps because it is considered trivial and uninteresting. Patients thoroughly reviewed our materials and gave useful feedback on things that we simply did not see as researchers. In particular they highlighted subtle differences in the terminology used and the need to give full consideration to how patients may interpret particular expressions in the context of their care, and how this could impact on patient well being.

These recommendations have already benefited our research procedures for Phase 1 of our study (which we acted on by submitting an amendment to the research ethics committee to make changes to the patient information sheet), and will further benefit our planned research in Phase 2.

Research ethics committees and sponsors typically have standard formats and requirements for information sheets, with which at least one participant was familiar. On this occasion we did not challenge these conventions. However, future research could explore shorter, simpler forms of the information sheets, removing the legal note at the bottom of the sheet, or more radical departures from the usual requirements and expectations of the Research Ethics Committee. Since doing the PPI workshop, our funders have requested that a funding acknowledgement and NIHR and Department of Health disclaimer is added to every information sheet, which may create further challenges in creating shorter forms and reflects different stakeholders’ interests and expectations.

### Informing research topics and sensitising researchers to patients’ experiences

The research questions that emerged from the workshop reflect the themes of the discussions we had around patients’ experiences of receiving intravenous infusions. This highlighted the situated nature of experiences of receiving medication intravenously in hospital. For example, patients might want more information but might not be given it, patients might have become expert in their own condition over a long period of time and feel confident in questioning medical professionals, and patients’ experiences can be influenced by staff attitude, staffing levels, device alarms, the environment, their own illness, their treatment and personal preferences. Similar to published findings we found a strong interplay of complex factors that seemed to impact patients’ experiences of the quality and safety of their infusion treatment (e.g. [20]). Indeed, it seemed difficult for patients to disentangle quality and safety issues [20].

Aspects of what was discussed have been referred to in the patient involvement in patient safety literature. For example, the attitude of staff affecting whether patients will speak up has been previously reported [6, 10, 14]. Also, it was clear that staff attitude played a critical role in the patient’s broad experience, not just their experience of safety. Participants expressed concern about undermining healthcare professionals by asking questions. This resonates with previous research on speaking up about safety (e.g. [6]). Workshop participants also expressed concern about patients raising negative issues with the researchers as it could compromise their relationship with staff (e.g. [6, 10, 14]).

In our ongoing research we will need to consider research into patient’s involvement in detecting, preventing and recovering from error [25], and patients’ reactions to alarms [19]. However, we are not aware of specific studies looking at the patient experience of intravenous infusion practices, and so the experiences shared in this PPI exercise provide a good foundation for our research.

### Challenges/limitations

The participants in this workshop described diverse experiences of different types of care as patients who had received intravenous infusions. However, we recognise that patients who take part in PPI activities may not be representative of the patient population in general. For example, one patient was a nurse and a number of participants had previous experience of involvement in research studies, research steering groups, or Research Ethics Committees in addition to their experiences as patients. Nonetheless, these patients provided rich detail about their patient experiences that contributed valuable insights, and they were able to view the problem from different angles.

Patients’ attitudes towards and expectations of healthcare services will be affected by their experience of harm. For example, a patient who has lost confidence in the healthcare service will be less likely to passively submit himself or herself to treatment. Experiencing harm was alluded to by at least one participant in the workshop, and both positive and negative experiences were reported more broadly. However, the direct relationship between experience of harm and the patient’s perspective of care was not explored in the workshop.

Furthermore, we asked patients to share their stories and experiences of intravenous infusions, and to provide feedback on the information sheets. Our data may therefore include a mixture of their own specific needs and their assumed needs of other patients. Further research could tease out any differences between these perspectives.

## Conclusion

There is a lack of literature on PPI for shaping patient safety research, either in showing how it can be conducted or the value it can bring. We have reported on three outcomes that show a clear contribution to our patient safety project: reviewing potential psychological risk to patient participants, simplifying patient information materials, and generating topics to pursue in research. There has also been great value in sensitizing the researchers to patients’ experiences in this area before we speak to patients at their bedsides on wards. These lessons could be of broader value to researchers in patient safety. For example, high profile advice has been published on how to handle ethical issues in patient safety research [28], but this does not include any form of PPI. This case study shows how PPI activities can positively contribute to this area.

## Abbreviations

NHS: National Health Service
PPI: patient and public involvement
WHO: World Health Organization
